# Determinants of pregnant women’s knowledge about influenza and the influenza vaccine: A large, single-centre cohort study

**DOI:** 10.1371/journal.pone.0236793

**Published:** 2020-07-31

**Authors:** Stéphanie Bartolo, Ophélie Mancel, Emilie Deliege, Sophie Carpentier, Rodrigue Dessein, Karine Faure, Damien Subtil

**Affiliations:** 1 Univ. Lille, CHU Lille, ULR 2694—METRICS: Évaluation des technologies de santé et des pratiques médicales, Lille, France; 2 Hôpital de Douai, Unité mères, Enfants et nouveau-nés, rue de Cambrai, Douai, France; 3 Univ. Lille, CHU Lille, Unité femmes, Mère et nouveaux nés, Lille, France; 4 Lille University, EA7366, Translational Research Host-Pathogen Relationships, Faculty of Medicine, Pôle Recherche, Lille, France; 5 Lille University, CHU Lille, Infectious Diseases Unit, rue Michel Polonowski, Lille, France; University of Oslo, NORWAY

## Abstract

**Introduction:**

Although influenza can lead to adverse outcomes during pregnancy, the level of influenza vaccine coverage among pregnant women remains very low. According to the literature, a high level of knowledge about influenza disease and the influenza vaccine is one of the main determinants of vaccination coverage. The objective of the present study was to describe pregnant women’s level of knowledge of these topics and to identify any corresponding determinants.

**Material and methods:**

A prospective, observational, hospital-based study of women having given birth in our university medical centre during the 2014–2015 influenza season. Data were collected through a self-questionnaire or extracted from medical records. Determinants of highest knowledge were identified using logistic regression.

**Results:**

Of the 2069 women included in the study, 827 (40%) did not know that influenza can lead to severe adverse outcomes for the mother, and 960 (46%) did not know about possible severe adverse outcomes for the baby. Two hundred and one women (9.8%) stated that the vaccine was “contraindicated” or “unnecessary” during pregnancy. Only 205 women (17%) had been vaccinated during a previous pregnancy. Determinants of the highest level of knowledge were age over 24, a high educational level, previous influenza vaccination, nulliparity, and the recommendation of vaccination by a healthcare professional.

**Conclusions:**

Recommending vaccination during pregnancy appears to increase knowledge about influenza and its vaccine among pregnant women.

## Introduction

Seasonal influenza is a common, contagious viral illness associated with elevated risks of morbidity and mortality in pregnant women [1]–even in women with no comorbidities [2]. Furthermore, a safe influenza vaccine is available; a review of 15 years of surveillance data in the United States (covering 750 million doses of influenza vaccine) did not highlight any safety problems for either the foetus or the mother [3–5]. Moreover, vaccination can reduce the incidence of influenza cases among vaccinated pregnant women by 70% [6] and among their infants by 63% [7]. The vaccine reduces the incidence of episodes of febrile respiratory illness by 29% [7], and maternal vaccination confers effective protection on the newborn [8]. This is why the seasonal influenza vaccination of pregnant women (regardless of gestational age) is recommended by World Health Organization [9], the American College of Obstetricians and Gynecologists, and the Centers for Disease Control and Prevention [10]. Despite these observations, influenza vaccination coverage among pregnant women remains very low: 7% in France (2015–2016) [11], 45% in England (2016–2017) [12], and 37% in the United States (2016–2017) [13].

Several studies have found that a good level of knowledge about influenza disease and the influenza vaccine is associated with higher rates of vaccine uptake during pregnancy [14–16]. However, few of these studies looked for determinants of high knowledge about these topics, and all had a small sample size [17, 18]. The objectives of the present study were to describe pregnant women’s levels of knowledge about these topics and to identify any corresponding determinants.

## Material and methods

During the 2014–2015 influenza season, we conducted a prospective, observational, single-centre study in a university medical centre’s level III maternity unit (Lille University Medical Centre, Lille, France). All women having received antenatal care during the 2014–2015 influenza vaccination campaign and having giving birth in our maternity unit between November 17^th^, 2014, and June 5^th^, 2015 were eligible for inclusion. All the included women gave their written, informed consent to participation. Each day, an investigator (OM or ED, both of whom are MDs in the unit) went to the unit to explain the study to the attending women and to collect their consent to participation in the study. We excluded women under the age of 18, those with a contraindication to influenza vaccination, and those who did not speak French. Some items of data were extracted from medical records. All the study participants were invited to fill out a self-questionnaire during their postpartum hospital stay (S1 and S2 Appendices).

The primary study outcome was a high level of knowledge about influenza and its vaccine, as assessed using a self-questionnaire based on that described by Yudin et al. [19]. We chose Yudin et al.’s questionnaire because it has been applied in several articles in this field [14, 15, 18]. To the best of our knowledge, none of the questionnaires used to probe levels of knowledge about vaccination has been psychometrically validated. Our questionnaire was drafted by a multidisciplinary expert group that included obstetricians, infectious disease specialists, general practitioners, and statisticians. We first tested the questionnaire on a randomly selected group of 10 pregnant women receiving antenatal care. Two questions were modified after this test. The questions covered the frequency of influenza infection, serious complications of influenza for mothers and infants, the frequency of complications of vaccination among mothers and their infants, and guidelines about vaccination during pregnancy. Before the study, we worked with the multidisciplinary expert group to create a "knowledge score" about influenza and its vaccine, based on the answers to 10 questions in the self-questionnaire. Each expert attributed a score of 0, 0.5 or 1 for each possible answer. The final scores for each answer were then decided on by consensus, and adopted as the scoring system. Depending on the type of question, the women were invited to answer “yes” or “no”, to check an answer, or to circle a number (from 0 to 9) that corresponded to their opinion. For example, the answers to the question “In your opinion, the influenza vaccine causes complications for the baby….” ranged from very rarely (0) to very frequently (9). A point was awarded for an answer below 3; no points were awarded for an answer of 3 or above (S3 Appendix). The overall score ranged from 0 to 10 points, a woman was considered to have a high level of knowledge if she achieved a score in the fourth quartile of the distribution (more than 6.0 out of 10, in the present study).

The following variables were considered as possible determinants of a high level of knowledge: the mother’s sociodemographic characteristics (age, educational level, and living with a partner or not); the mother’s medical history before pregnancy (pre-existing comorbidities for which influenza vaccination is recommended by the French guidelines [20], having being vaccinated against influenza prior to the current pregnancy, the number of previous deliveries, and any history of preterm delivery (before 34 weeks of gestation)); the characteristics of the current pregnancy (smoking status, and obstetrical complications such as gestational diabetes, gestational hypertension, pre-eclampsia, HELLP syndrome, infections, and foetal growth restriction); prenatal care (the healthcare professional providing the prenatal care: a gynaecologist/obstetrician, a general practitioner, a hospital midwife, or a private midwife), and the profession of the healthcare professional who recommended vaccination (i.e. the source of information about the vaccine).

Data on prenatal care and on the mother’s knowledge about influenza and its vaccine were extracted from the self-questionnaire. Data on the mother’s sociodemographic characteristics, the mother’s medical characteristics before pregnancy, and the current pregnancy’s characteristics were extracted from medical records.

### Statistical analyses

Determinants associated with a high level of knowledge were identified in bivariate and multivariate analyses. Variables significantly associated with a high level of knowledge in the bivariate analysis (p<0.20) were included in the multivariate model. Percentages were compared in a chi-squared test or (depending on the number of individuals) Fisher's exact test. We calculated adjusted odds ratios (aORs) with their 95% confidence interval (CI). The threshold for statistical significance was set to p<0.05. All statistical analyses were performed with STATA software (version 13.0.0, StataCorp LP, College Station, TX, USA).

### Ethical approval

The study’s objectives and procedures were approved by the local independent ethics committee (CEROG, Lille, France; reference: OBS 2014-11-01).

## Results

**Fig 1:**
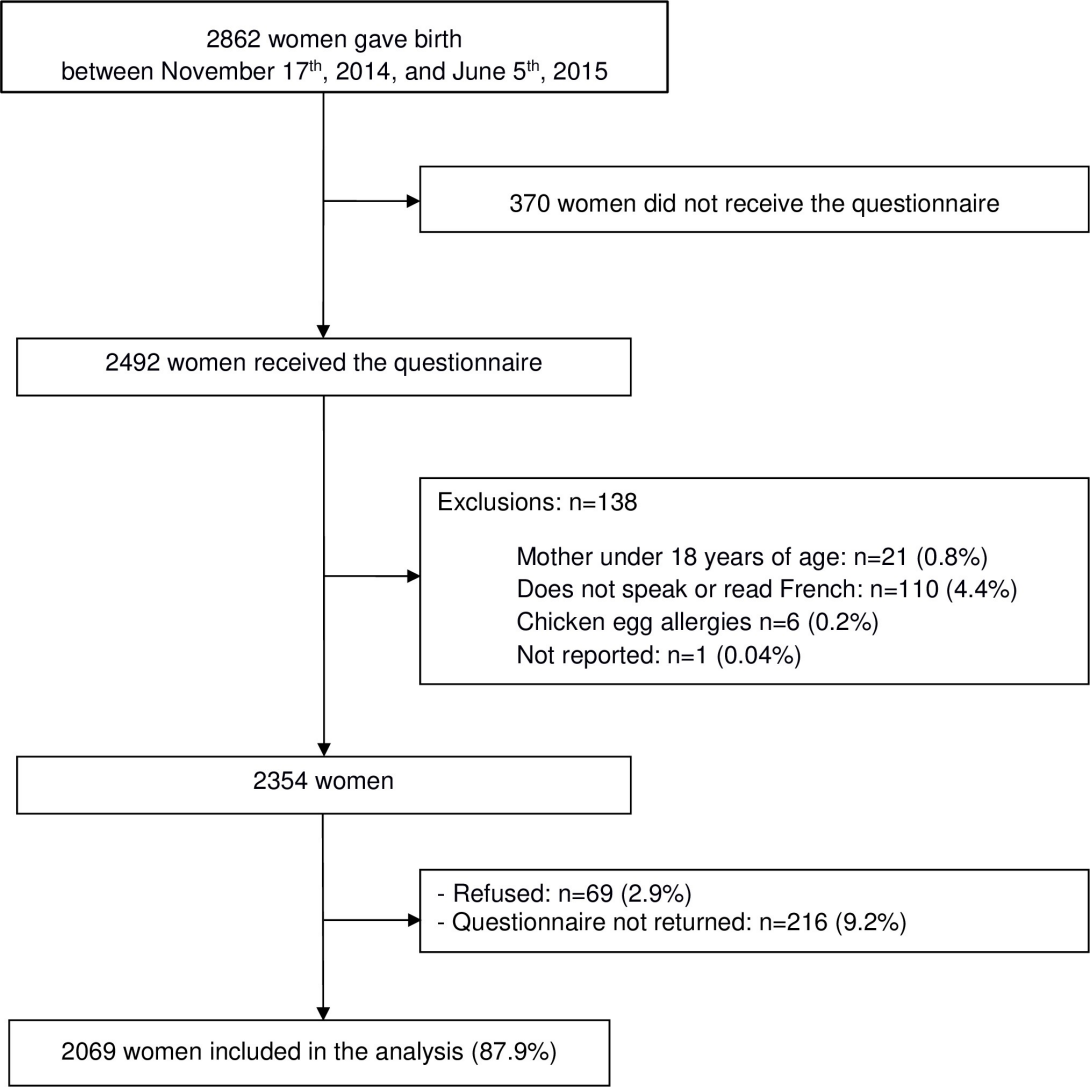
Study flow chart.

Of the 2862 women having giving birth during the study period, 370 (12.9%) did not receive the study questionnaire because the investigator did not attend the maternity unit during the women’s stay, and 138 (5.5%) were excluded (Fig 1). Next, 69 of the 2358 eligible women (2.9%) refused to participate, and 216 (9.2%) did not return the study questionnaire. Hence, 2069 women of the 2354 eligible women were included in the study (87.9%).

**Fig 2:**
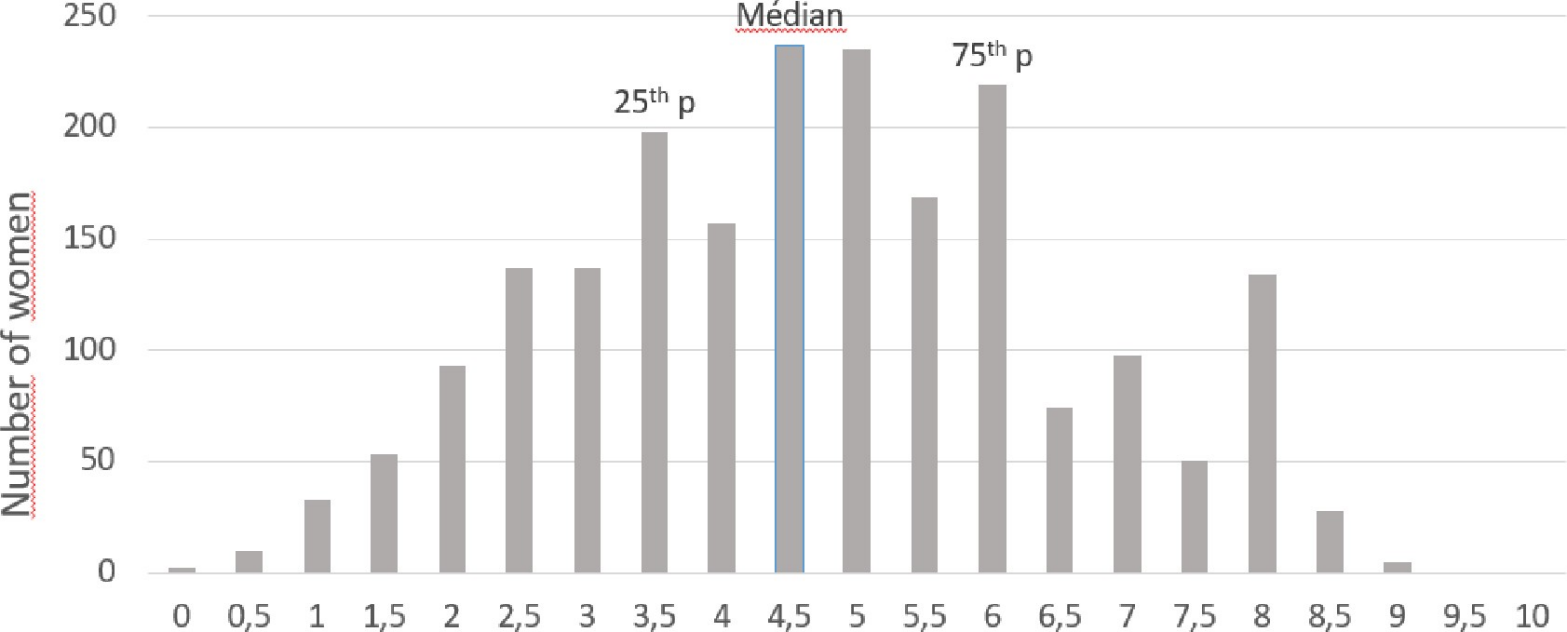
Distribution of Knowledge scores.

Our data on the women’s knowledge about influenza disease and the influenza vaccine are summarized in Table 1. Firstly, 827 of the women (40.1%) did not know that influenza can lead to serious complications for the mother, and 960 (46.6%) did not know that influenza can lead to be serious complications for the baby. Secondly, 201 ‬women (9.8%) considered the vaccine to be “contraindicated” or “unnecessary” during pregnancy. Only 205 women (17.4%) had been vaccinated during a previous pregnancy. 1391 women (69.2%) listed a healthcare professional as their main source of information. 870 (45.7%) of the women estimated that the frequency of adverse reactions to the influenza vaccine was “common” or “very common” for mothers, and 906 (48.0%) estimated that the frequency of adverse reactions to the vaccine was “common” or “very common” for babies.‬‬‬‬‬‬ The distribution of the knowledge score data is shown in Fig 2. The median score [interquartile range] was 4.5 [3.5–6.0]). A total of 608 women (29.4%) had the highest level of knowledge‬‬‬‬‬‬‬‬‬ (i.e. a knowledge score in the fourth quartile, corresponding to 6 and over).‬‬‬‬‬‬‬

**Table 1:** Women’s level of knowledge about influenza and its vaccine during pregnancy.

		n	%
**Perceived frequency of influenza in the general population** (n = 2020)^‡^			
	Very rare (0–2) *	79	3.9
	Rare (3–4) *	353	17.5
	Common (5–6)	1124	55.6
	Very common (7–9)	464	23.0
**Influenza can induce serious complications for the mother** (n = 2060)			
	Yes*	1233	59.9
	No.	147	7.1
	I don’t know	680	33.0
**Influenza can induce serious complications for the baby** (n = 2062)			
	Yes*	1102	53.4
	No.	134	6.5
	I don’t know	826	40.1
**Utility of influenza vaccination during pregnancy** (n = 2055)			
	Contraindicated	107	5.2
	Unnecessary	94	4.6
	Might be useful *	875	42.6
	Definitely useful*	979	47.6
**Recommendation of vaccination during pregnancy** (n = 2064)			
	Either not obligatory or not recommended	247	12.0
	Obligatory *	60	2.9
	Recommended *	1525	73.9
	I don’t know	232	11.2
**Previous influenza vaccination** (n = 2065)			
	No	1245	60.3
	Yes, outside pregnancy	554	26.8
	Yes, during a previous pregnancy (17.4% of the 1180 multiparous women)	205	9.9
	I don’t know	61	3.0
**Sources of information about influenza vaccination** (each source could be named on the questionnaire) (n = 2010)			
	Healthcare professionals (as well as other sources, potentially) ^a^	1391	69.2
	Other sources only (i.e. not healthcare professionals)^b^	619	30.8
**Perceived frequency of adverse reactions to the vaccine among mothers** (n = 1902) ^‡^			
	Very rare (0–2)*	525	27.6
	Rare (3–4)	507	26.7
	Common (5–6)	706	37.1
	Very common (7–9)	164	8.6
**Perceived frequency of adverse reactions to the vaccine among babies** (n = 1887) ^‡^			
	Very rare (0–2)*	531	28.1
	Rare (3–4)	420	22.3
	Common (5–6)	664	35.2
	Very common (7–9)	242	12.8

* considered to be a correct answer by the expert group

‡ the responses are adapted from a number scale from 0 to 9.

^a^ Either healthcare professionals only, or healthcare professionals and other sources, such as the media, discussion groups, family & friends, health authorities, etc.

^b^ All possible sources (the media, discussion groups, family & friends, health authorities, etc.) other than healthcare professionals.

In the bivariate analysis, the factors associated with the highest level of knowledge were older maternal age, non-smoking status, previous influenza vaccination, a low number of children, prenatal care provided by an obstetrician in hospital, a recommendation of vaccination by a healthcare professional, and having received information from one or more healthcare professionals (Table 2).

**Table 2:** Factors associated with the highest level of knowledge among women about influenza and its vaccine during pregnancy: A bivariate analysis.

		n/N*	%	p
**Total**		608/2069	29.4	
**Age (years)**				<0.001
	≤ 24	62/314	19.7	
	25–29	195/653	29.9	
	30–35	220/689	31.9	
	≥35	131/412	31.8	
**Educational level**				<0.001
	Primary education	11/73	15.1	
	Secondary or technical education	71/394	18.0	
	Higher education	525/1600	32.8	
**Living with a partner**				0.006
	Yes	554/1820	30.4	
	No	54/247	21.9	
**Smoked during pregnancy**				0.007
	Yes	108/444	24.3	
	No	500/1619	30.9	
**Number of previous deliveries**				0.002
	0	283/887	31.9	
	1	217/711	30.5	
	≥ 2	108/469	23.0	
**History of preterm delivery (<34 weeks)**				0.35
	Yes	19/77	24.7	
	No	589/1990	29.6	
**Pre-existing comorbidities for which influenza vaccination is recommended**				0.60
	Yes	147/485	30.3	
	No	457/1573	29.1	
**Previous influenza vaccination**				<0.001
	Yes, outside pregnancy	231/554	41.7	
	Yes, during a previous pregnancy	92/205	44.9	
	No	271/1245	21.8	
**Obstetric complications**^**a**^				0.13
	Yes	227/822	27.6	
	No	381/1242	30.7	
**Healthcare professional providing prenatal care**				0.26
	Hospital staff midwife	257/904	28.4	
	Hospital staff physician	243/741	32.8	
	Assistant chief resident	37/130	28.5	
	Intern	54/187	28.9	
**Vaccination recommended by a healthcare professional**				<0.001
	Yes	522/1522	34.3	
	No	86/531	16.2	
**Healthcare professional recommending vaccination**				0.002
	Hospital staff midwife	165/584	28.2	
	Private midwife	26/66	39.4	
	General practitioner	80/202	39.6	
	Gynaecologist/obstetrician	214/576	37.2	
**Sources of information** (each source could be named on the questionnaire)				<0.001
	Healthcare professionals (as well as other sources, potentially)^b^	450/1391	32.4	
	Other sources only (not healthcare professionals)^c^	151/619	24.4	

* The proportion of women with the highest level of knowledge in each subgroup; n: the number of women with the highest level of knowledge in the subgroup; N: the total number of women in the subgroup.

^a^ Gestational diabetes, gestational hypertension, pre-eclampsia, HELLP syndrome, infections, or another reason.

^b^ Either healthcare professionals only, or healthcare professionals and other sources, such as the media, discussion groups, family & friends, health authorities, etc.

^c^ All possible sources (the media, discussion groups, family & friends, health authorities, etc.) other than healthcare professionals.

In the multivariate logistic regression analysis (Table 3), the variables significantly associated with the highest level of knowledge about influenza and its vaccine were age over 24, a high educational level, previous influenza vaccination, nulliparity, and a recommendation of vaccination by a healthcare professional.

**Table 3:** Factors associated with women’s knowledge about influenza and its vaccine during pregnancy: A multivariate analysis (N = 1983).

		aOR^a^	95%CI^b^	p
**Age (years)**				0.02
	≤ 24	1		
	25–29	1.52	1.1–2.2	
	30–35	1.80	1.2–2.6	
	≥35	1.76	1.2–2.7	
**Educational level**				<0.001
	Primary education	1		
	Secondary or technical education	1.3	0.6–2.7	
	Higher education	2.3	1.1–4.7	
**Number of previous deliveries**				0.006
	≥2	1		
	1	1.5	1.1–2.0	
	0	1.6	1.2–2.2	
**Previous influenza vaccination**				<0.001
	No	1		
	Yes, outside pregnancy	2.5	2.0–3.1	
	Yes, during a previous pregnancy	2.9	2.1–4.0	
**Vaccination recommended by a healthcare professional**				<0.001
	No	1		
	Yes	2.4	1.8–3.1	

^a^ The adjusted odds ratio for a high level of knowledge about influenza and its vaccine, determined in a multivariate logistic regression analysis. Variables not significantly associated (p>0.05) with knowledge about influenza and its vaccine (living with a partner, smoking during pregnancy, obstetric complications, and sources of information) are not presented.

^b^ CI: confidence interval.

## Discussion

Our results show that a large proportion of pregnant women having given birth in a university medical centre in France were not sufficiently knowledgeable about influenza and its vaccine. Indeed, about half of the pregnant women did not know that influenza can lead to serious complications for them and/or for their babies. With a view to improving vaccine coverage in this population, the only easily actionable factor associated with a higher level of knowledge about influenza and its vaccine was the recommendation of vaccination during pregnancy by a healthcare professional.

Our research was observational and so was affected by the inherent limitations of this type of study. Furthermore, the study was carried out during the 2014–2015 influenza season; in the middle of the vaccination campaign, the vaccine’s efficacy was challenged in the French media. This may have influenced the vaccination rate and/or the women’s answers in the questionnaire.

Although the women’s level of knowledge was far from optimal, we found that the vaccination rate among the study participants was 35%; this value is higher than the national average for France (7%, currently) [11] but is similar to the rates observed in other countries: 45% in England in 2017 [12] and 37% in the United States in 2017, for example [13]. However, we did our best to reduce the risk of bias: our prospective study included a high proportion (87.1%) of the women who gave birth in our hospital during the study period, and the questionnaire response rate was high (87.9%). Moreover, we chose to administer a self-questionnaire so that the participants’ answers were not influenced by the medical staff.

In the present study, a high level of knowledge about influenza and its vaccine was defined as knowing that influenza infection (i) is frequent and contagious, (ii) can lead to rare but serious complications for pregnant women and babies, and (iii) can be mitigated by a readily available, effective, guideline-recommended vaccine. We found that 40.1% of the pregnant women did not know that influenza could cause serious complications for the mother, and 46.6% did not know that influenza could cause serious complications for the baby. These values of over 40% were higher than those reported in the United States [21], Switzerland [14] and Korea [15], where prospective studies found that only 20% of women did not know that influenza could have serious complications during pregnancy for the mother and her baby.

In the present study, the majority of the participants (90.2%) believed that the vaccine “might be useful” or “definitely useful”. Our results differed from those observed in an Italian study, in which only 41% of pregnant women thought that a vaccine could protect pregnant women against influenza [17]. The same result was found in the French Vaccinoscopie® study in 2014; only a third of the 300 surveyed women with a child aged 12 months or younger thought that it was “rather important” or “very important” to be protected against influenza during pregnancy [22]. The vaccine’s perceived utility might be counterbalanced by a fear of adverse reactions [23]. In the literature, 30% to 50% of pregnant women thought that the vaccine might induce influenza or influenza-like symptoms [24], and 15% feared that the vaccine could cause foetal defects [25] or premature birth [15]. More generally, 46% of the women in the US study considered that vaccination during pregnancy was not safe [21].

Although higher vaccination rates are generally found among women with a better level knowledge about influenza and its vaccine [14–16, 18, 24], the present study is one of the few to have looked for factors associated with a high level of knowledge [17, 18, 21]. In a study in Saudi Arabia, Mayet et al. interviewed 998 women about the influenza vaccine; working women and those with at least one child had a higher level of knowledge. Napolitano et al.’s study of 372 pregnant women in Italy found that a better level of knowledge was associated with older age, a higher educational level, and a high-risk pregnancy [17]. In the present study, we found that age over 24, a high educational level, previous influenza vaccination, nulliparity, and having been recommended influenza vaccination during pregnancy were significantly associated with greater knowledge about influenza and its vaccine [17]. Unsurprisingly, Mayet et al. and Napolitano et al. also found that a higher educational level was associated with a higher level of knowledge about influenza [17]. Furthermore, women having been previously vaccinated against influenza have the highest level of knowledge about influenza and its vaccine. This is also true outside pregnancy [14, 24, 26].

Among the factors found here to be associated with the highest level of knowledge about influenza and its vaccine among pregnant women, the recommendation of vaccination during pregnancy is the only one that could be promoted by a public health initiative. We are not aware of a study that has evaluated the specific impact of recommending vaccination on a woman’s level of knowledge during pregnancy. However, several studies have evidenced a direct, statistically significant association between a recommendation of vaccination and subsequent vaccination [14, 24]. In the present study, influenza vaccination had been recommended to 73.6% of the participants. Only a third of these women had a high level of knowledge, even though 67.2% of the study population reported that healthcare professionals constituted their main source of information about influenza and its vaccine. Similarly, 65% of American women considered healthcare professionals to be the most important, trusted source of information during their pregnancy [27]. Hence, it seems possible that the suboptimal level of knowledge in our study population might be due to a lack of knowledge among healthcare professionals–some of whom may not be convinced of the value of vaccination against influenza. Indeed, awareness of an elevated risk of influenza-induced deaths among pregnant women was heightened by the H1N1 influenza pandemic in 2009 [28]. Moreover, scepticism about vaccination among healthcare professionals in France has notably increased since the 1990s [29].

On the basis of our present results, recommending vaccination during pregnancy appears to increase knowledge about influenza and its vaccine among pregnant women. An evaluation of healthcare providers’ knowledge and attitudes regarding influenza vaccination is warranted.

## Supporting information

S1 Appendix The self-administered questionnaire in French. (DOC)

S2 Appendix The self-administered questionnaire in English. (DOCX)

S3 Appendix The scoring system for the “knowledge score”. (DOCX)

S1 Data (XLSX)

## References

[1] Vaccines against influenza WHO position paper–November 2012. *Relevé Épidémiologique Hebd Sect Hygiène Secrétariat Société Nations Wkly Epidemiol Rec Health Sect Secr Leag Nations* 2012; 87: 461–476.

[2] Anselem O, Floret D, Tsatsaris V, et al [Influenza infection and pregnancy]. *Presse Médicale Paris Fr 1983* 2013; 42: 1453–1460. 10.1016/j.lpm.2013.01.064 23683385

[3] Vellozzi C, Burwen DR, Dobardzic A, et al Safety of trivalent inactivated influenza vaccines in adults: background for pandemic influenza vaccine safety monitoring. *Vaccine* 2009; 27: 2114–2120. 10.1016/j.vaccine.2009.01.125 19356614

[4] Louik C, Kerr S, Van Bennekom CM, et al Safety of the 2011–12, 2012–13, and 2013–14 seasonal influenza vaccines in pregnancy: Preterm delivery and specific malformations, a study from the case-control arm of VAMPSS. *Vaccine* 2016; 34: 4450–4459. 10.1016/j.vaccine.2016.06.078 27452865

[5] Chambers CD, Johnson DL, Xu R, et al Safety of the 2010–11, 2011–12, 2012–13, and 2013–14 seasonal influenza vaccines in pregnancy: Birth defects, spontaneous abortion, preterm delivery, and small for gestational age infants, a study from the cohort arm of VAMPSS. *Vaccine* 2016; 34: 4443–4449. 10.1016/j.vaccine.2016.06.054 27449682

[6] Håberg SE, Trogstad L, Gunnes N, et al Risk of fetal death after pandemic influenza virus infection or vaccination. *N Engl J Med* 2013; 368: 333–340. 10.1056/NEJMoa1207210 23323868 PMC3602844

[7] Zaman K, Roy E, Arifeen SE, et al Effectiveness of maternal influenza immunization in mothers and infants. *N Engl J Med* 2008; 359: 1555–1564. 10.1056/NEJMoa0708630 18799552

[8] Nunes MC, Cutland CL, Jones S, et al Duration of Infant Protection Against Influenza Illness Conferred by Maternal Immunization: Secondary Analysis of a Randomized Clinical Trial. *JAMA Pediatr* 2016; 170: 840–847. 10.1001/jamapediatrics.2016.0921 27380464

[9] World Health Organization. Weekly epidemiological record Relevé épidémiologique hebdomadaire. 2012; 461–476. 23210147

[10] Committee on Obstetric Practice and Immunization Expert Work Group, Centers for Disease Control and Prevention’s Advisory Committee on Immunization, United States, American College of Obstetricians and Gynecologists. Committee opinion no. 608: influenza vaccination during pregnancy. *Obstet Gynecol* 2014; 124: 648–651. 10.1097/01.AOG.0000453599.11566.11 25162283

[11] Blondel B, Coulm B, Bonnet C, et al Trends in perinatal health in metropolitan France from 1995 to 2016: Results from the French National Perinatal Surveys. *J Gynecol Obstet Hum Reprod* 2017; 46: 701–713. 10.1016/j.jogoh.2017.09.002 29031048

[12] Surveillance_of_influenza_and_other_respiratory_viruses_in_the_UK_2016_to_2017.pdf, https://www.gov.uk/government/uploads/system/uploads/attachment_data/file/613493/Surveillance_of_influenza_and_other_respiratory_viruses_in_the_UK_2016_to_2017.pdf (accessed 11 June 2017).

[13] Ding H, Black CL, Ball S, et al Influenza Vaccination Coverage Among Pregnant Women—United States, 2016–17 Influenza Season. *MMWR Morb Mortal Wkly Rep* 2017; 66: 1016–1022. 10.15585/mmwr.mm6638a2 28957044 PMC5657675

[14] Blanchard-Rohner G, Meier S, Ryser J, et al Acceptability of maternal immunization against influenza: the critical role of obstetricians. *J Matern Fetal Neonatal Med* 2012; 25: 1800–1809. 10.3109/14767058.2012.663835 22339083

[15] Ko HS, Jo YS, Kim YH, et al Knowledge, attitudes, and acceptability about influenza vaccination in Korean women of childbearing age. *Obstet Gynecol Sci* 2015; 58: 81–89. 10.5468/ogs.2015.58.2.81 25798420 PMC4366874

[16] Yudin MH. Risk management of seasonal influenza during pregnancy: current perspectives. *Int J Womens Health* 2014; 6: 681–689. 10.2147/IJWH.S47235 25114593 PMC4122531

[17] Napolitano F, Napolitano P, Angelillo IF. Seasonal influenza vaccination in pregnant women: knowledge, attitudes, and behaviors in Italy. *BMC Infect Dis* 2017; 17: 48 10.1186/s12879-016-2138-2 28068918 PMC5223411

[18] Mayet AY, Al-Shaikh GK, Al-Mandeel HM, et al Knowledge, attitudes, beliefs, and barriers associated with the uptake of influenza vaccine among pregnant women. *Saudi Pharm J*. 10.1016/j.jsps.2015.12.001 28223865 PMC5310150

[19] Yudin MH, Salaripour M, Sgro MD. Pregnant women’s knowledge of influenza and the use and safety of the influenza vaccine during pregnancy. *J Obstet Gynaecol Can JOGC J Obstet Gynecol Can JOGC* 2009; 31: 120–125. 10.1016/s1701-2163(16)34095-6 19327210

[20] calendrier_vaccinal_2016.pdf, http://solidarites-sante.gouv.fr/IMG/pdf/calendrier_vaccinal_2016.pdf (accessed 12 June 2017).

[21] Chamberlain AT, Seib K, Ault KA, et al Factors Associated with Intention to Receive Influenza and Tetanus, Diphtheria, and Acellular Pertussis (Tdap) Vaccines during Pregnancy: A Focus on Vaccine Hesitancy and Perceptions of Disease Severity and Vaccine Safety. *PLoS Curr*; 7 Epub ahead of print 25 February 2015. 10.1371/currents.outbreaks.d37b61bceebae5a7a06d40a301cfa819 25789203 PMC4353696

[22] Gaudelus, J, Martinot, A, Denis, F, et al. Prévention vaccinale chez la femme enceinte: les données françaises.

[23] Yuen CYS, Tarrant M. Determinants of uptake of influenza vaccination among pregnant women–A systematic review. *Vaccine* 2014; 32: 4602–4613. 10.1016/j.vaccine.2014.06.067 24996123

[24] Tong A, Biringer A, Ofner-Agostini M, et al A cross-sectional study of maternity care providers’ and women’s knowledge, attitudes, and behaviours towards influenza vaccination during pregnancy. *J Obstet Gynaecol Can JOGC J Obstétrique Gynécologie Can JOGC* 2008; 30: 404–410. 10.1016/s1701-2163(16)32825-0 18505664

[25] Maurici M, Dugo V, Zaratti L, et al Knowledge and attitude of pregnant women toward flu vaccination: a cross-sectional survey. *J Matern Fetal Neonatal Med* 2015; 0: 1–4. 10.3109/14767058.2015.1118033 26555821

[26] Freund R, Le Ray C, Charlier C, et al Determinants of Non-Vaccination against Pandemic 2009 H1N1 Influenza in Pregnant Women: A Prospective Cohort Study. *PLoS ONE*; 6 Epub ahead of print 14 June 2011. 10.1371/journal.pone.0020900 21695074 PMC3114856

[27] Beel ER, Rench MA, Montesinos DP, et al Knowledge and attitudes of postpartum women toward immunization during pregnancy and the peripartum period. *Hum Vaccines Immunother* 2013; 9: 1926–1931. 10.4161/hv.25096 PMC3906358 23782490

[28] Influenza Investigators ANZIC, Webb SAR, Pettilä V, et al Critical care services and 2009 H1N1 influenza in Australia and New Zealand. *N Engl J Med* 2009; 361: 1925–1934. 10.1056/NEJMoa0908481 19815860

[29] Yaqub O, Castle-Clarke S, Sevdalis N, et al Attitudes to vaccination: a critical review. *Soc Sci Med 1982* 2014; 112: 1–11. 10.1016/j.socscimed.2014.04.018 24788111

