# Supplementary material for: Determinants of pregnant women’s knowledge about influenza and the influenza vaccine: A large, single-centre cohort study
Source: PLoS One. 2020 Jul 31;15(7):e0236793. doi: 10.1371/journal.pone.0236793 (PMC7394385; doi:10.1371/journal.pone.0236793)
Supplement: S2 Appendix — (DOCX) [file pone.0236793.s002.docx]

G2014/1/ _ _ _ _

Family name

First name

Date of birth

G2014/1/ _ _ _ _

1. Before you can participate in this study, we need to know some information:

- Are you 18 or over? 🞎 yes 🞎 no
- Can you speak French? 🞎 yes 🞎 no
- Can you read French? 🞎 yes 🞎 with difficulty 🞎 not at all
- Are you allergic to egg proteins? 🞎 no 🞎 yes

Answer the 4 questions but THEN STOP if you have checked any of the gray boxes in question (a)

- Do you wish to participate in this study? 🞎 yes 🞎 no

1. What is your educational level?

- primary 🞎 secondary or technical 🞎 higher

1. When did you start receiving antenatal care at Jeanne de Flandre Hospital?

- during the 1^st^ trimester 🞎 during the 2^nd^ trimester 🞎 during the 3^rd^ trimester

1. What is the name of the person who is looking after you at Jeanne de Flandre Hospital? ……………………………….
2. In your opinion, influenza is a disease that is:
   *(on each line, circle the number that corresponds to your opinion)*

Very rare 0 1 2 3 4 5 6 7 8 9 Very common

Never serious 0 1 2 3 4 5 6 7 8 9 Always serious

1. Do you think that influenza during pregnancy can cause serious complications for the mother?

- Yes 🞎 No 🞎 I don't know
- If you answered “yes”, can you give an example? ……………………………………………………………………………

1. Do you think that influenza during pregnancy can cause serious complications for the baby?

- Yes 🞎 No 🞎 I don't know
- If you answered “yes”, can you give an example? ……………………………………………………………………………

1. In your opinion, vaccination against influenza during pregnancy is:

- Contraindicated 🞎 Unnecessary 🞎 Might be useful 🞎 Definitely useful

1. In your opinion, vaccination against influenza during pregnancy is:

- Obligatory 🞎 Neither obligatory nor recommended
- Recommended by the health authorities 🞎 I don't know

1. Have you ever been vaccinated against influenza?

- Yes, outside pregnancy 🞎 No
- Yes, during a previous pregnancy 🞎 I don't know

1. What were your sources of information regarding influenza vaccination? (tick all that apply)

- Healthcare professionals (physicians, midwifes, nurses, pharmacists, etc.)
- Media (radio, television, newspapers, magazines, internet, advertising posters, etc.)
- Discussion forums (chats, groups, etc.)
- Family and friends
- The official [French] health authorities (HAS, INPES, ANSM, etc.)
- Other sources: ………………………………………………………………………………………………………………………..............

1. Which source prompted your decision to get vaccinated (or to not get vaccinated)? …………………………..

**PLEASE GO TO THE NEXT PAGE**

1. During your current pregnancy, has vaccination against influenza been recommended to you?

- Yes 🞎 No
- If so, who recommended it to you?

🞎 A gynecologist/obstetrician at Jeanne de Flandre Hospital 🞎 A general practitioner

🞎 A midwife at Jeanne de Flandre Hospital 🞎 A private practice midwife

🞎 Other: …………………………………………………………………………………………………………………………………..

1. During your pregnancy, were you given the form for the reimbursement of influenza vaccination by the social security (a free voucher)?

- Yes 🞎 No

1. Have you been vaccinated against influenza during this pregnancy?

- Yes 🞎 No 🞎 I don't know

**☞If you answered “yes”**,

- Who vaccinated you? ………………………………………………………………………………………………………
- When during pregnancy were you vaccinated?
- 1^st^ trimester (0 -3 months) 🞎 2^nd^ trimester (4-6 months) 🞎 3^rd^ trimester (7-9 months)
- What made you decide to get vaccinated? (tick all that apply)
- The vaccine protects me
- The vaccine protects my baby
- I received enough information about the benefits of influenza vaccination
- I am generally in favour of vaccines
- Influenza vaccination is free of charge
- Another reason: …………………………………………………………………………………………………………………….

**☞If you answered “no”,**

- What were your reservations? (tick all that apply)
- I didn’t know that there was a vaccine
- I was scared for my baby's health
- I was scared for my health
- I did not have enough information about the benefits and risks
- I am generally against vaccines
- Another reason: …………………………………………………………………………………………………………………….

1. Which person helped you most to make your choice?

🞎 General practitioner 🞎 Private practice obstetrician/midwife 🞎 Hospital obstetrician/midwife 🞎 Friend/family

🞎 Other

1. In your opinion, the influenza vaccine causes complications for the mother:

*(circle the number that corresponds to your opinion)*
Very rare 0 1 2 3 4 5 6 7 8 9 Very common

Never serious 0 1 2 3 4 5 6 7 8 9 Always serious

In your opinion, the influenza vaccine causes complications for the baby:
*(circle the number that corresponds to your opinion)*
Very rare 0 1 2 3 4 5 6 7 8 9 Very common

Never serious 0 1 2 3 4 5 6 7 8 9 Always serious

1. If you have not yet been vaccinated, would you like to do it now?

🞎 Yes 🞎 No

**THANK YOU FOR PARTICIPATING!**
