# Supplementary material for: Determinants of pregnant women’s knowledge about influenza and the influenza vaccine: A large, single-centre cohort study
Source: PLoS One. 2020 Jul 31;15(7):e0236793. doi: 10.1371/journal.pone.0236793 (PMC7394385; doi:10.1371/journal.pone.0236793)
Supplement: S3 Appendix — (DOCX) [file pone.0236793.s003.docx]

SCORING SYSTEM FOR THE QUESTIONNAIRE

1. In your opinion, influenza is a disease that is: (on each line, circle the number that corresponds to your opinion)

Very rare 0 1 2 3 4 5 6 7 8 9 Very common

*If the answer is <5: 1 point.*

Never serious 0 1 2 3 4 5 6 7 8 9 Always serious

*If the answer is <5: 1 point.*

1. Do you think that influenza during pregnancy can cause serious complications for the mother?

*If the answer is “yes”: 1 point.*

1. Do you think that influenza during pregnancy can cause serious complications for the baby?

*If the answer is “yes”: 1 point.*

1. In your opinion, vaccination against influenza during pregnancy is:

*If the answer is “Might be useful”: 0.5 points.*

*If the answer is “Definitely useful”: 1 point.*

1. In your opinion, vaccination against influenza during pregnancy is :

*If the answer is “Obligatory”: 0.5 points.*

*If the answer is “Recommended”: 1 point.*

1. In your opinion, the influenza vaccine causes complications for the mother:

*(circle the number that corresponds to your opinion)*

Very rare 0 1 2 3 4 5 6 7 8 9 Very common

*If the answer is 5, 6 or 7: 1 point.*

Never serious 0 1 2 3 4 5 6 7 8 9 Always serious

*If the answer is <3: 1 point*

1. In your opinion, the influenza vaccine causes complications for the baby:

*(circle the number that corresponds to your opinion)*

Very rare 0 1 2 3 4 5 6 7 8 9 Very common

*If the answer is <3: 1 point.*

Never serious 0 1 2 3 4 5 6 7 8 9 Always serious

*If the answer is <3: 1 point*
